# Changes in tonsillolith characteristics detected in a follow-up CT study

**DOI:** 10.1186/s12903-021-01426-1

**Published:** 2021-02-16

**Authors:** Kouhei Yamashita, Masafumi Oda, Tatsurou Tanaka, Ikuko Nishida, Nao Wakasugi-Sato, Shinobu Matsumoto-Takeda, Manabu Habu, Teppei Sago, Osamu Takahashi, Hiroki Tsurushima, Shiro Tabe, Taishi Otani, Daigo Yoshiga, Masaaki Sasaguri, Takaaki Joujima, Yuichi Miyamura, Yasuhiro Morimoto

**Affiliations:** 1 Division of Oral and Maxillofacial Radiology, Kyushu Dental University, 2-6-1 Manazuru, Kokurakita-ku, Kitakyushu, 803-8580 Japan; 2 Division of Developmental Stomatognathic Function Science, Kyushu Dental University, Kitakyushu, Japan; 3 Division of Maxillofacial Surgery, Kyushu Dental University, Kitakyushu, Japan; 4 Division of Dental Anesthesiology, Kyushu Dental University, Kitakyushu, Japan; 5 Division of Oral Medicine, Kyushu Dental University, Kitakyushu, Japan

**Keywords:** Tonsilloliths, CT, Follow up, Change, Characteristics

## Abstract

**Background:**

Tonsilloliths are related clinically to halitosis and tonsillar abscess. However, the dynamics of tonsilloliths over time are unknown. The aim of the study was to evaluate change in the characteristics of tonsilloliths in a time-dependent fashion by follow-up computed tomography (CT).

**Methods:**

Tonsilloliths were analyzed in 326 CT scan pair sets of initial and at least two follow-up CT examinations of patients with whole palatine tonsils and various diseases of the oral and maxillofacial regions.

**Results:**

Over the follow-up period, 12.1% of tonsilloliths disappeared. Approximately 26.1% of tonsilloliths changed in size during follow-up, mostly increasing in size. In tonsilloliths that showed enlargement, the mean (± standard deviation) growth rate was 0.61 ± 0.41 mm per year. Approximately 37.3% of tonsilloliths changed position during the follow-up period; of these, movement was toward the respiratory tract in 92% at a mean rate of − 1.38 ± 1.59 mm per year. The calcification levels of almost all tonsilloliths showed dynamic change: HU number increased in 84.3% and decreased in 12.7% of tonsilloliths over the follow-up period. The mean rate of HU increase was 63.8 ± 96.3 HU/year, and the mean rate of HU decrease was − 38.4 ± 66.8 HU/year.

**Conclusions:**

The calcification levels of all tonsilloliths showed dynamic fluctuation, and a tendency for excretion of tonsilloliths from the body. Their dynamics over time suggest that tonsilloliths may be in a permanently active phase which functions to remove foreign matter.

## Background

Tonsilloliths are oropharyngeal concretions that form within the palatine tonsillar crypt in reaction to a foreign nidus such as bacteria or organic debris. As it has been suggested that tonsilloliths are related clinically to halitosis and tonsillar abscess, these structures are of interest in the field of dentistry [1–8]. Tonsilloliths can be visualized on digital panoramic radiographs [1, 9]. Recent CT studies have revealed that the prevalence of tonsilloliths is much higher than previously thought, and have reported a detection rate on CT of 46.1% [1, 10–13]. In addition, characteristics of tonsilloliths such as the degree of calcification and size have been described [1]. Because their presence is age-dependent, we might expect to see dynamic change in tonsilloliths. However, all previous reports have described cross-sectional studies, and there has been no evaluation of the changing characteristics of tonsilloliths with time.

The present longitudinal study evaluated the number of tonsilloliths and their size, position, and degree of calcification in a time-dependent fashion by follow-up CT, with the aim of elucidating tonsillolith activity and investigating their excretion from the body as a system of removal of foreign matter.

## Methods

The subjects were 326 patients with whole palatine tonsils (160 males, 166 females; mean age, 63.3 ± 17.3 years; age range, 11–93 years) and who underwent CT of the oral or maxillofacial region for investigation of the following: malignant tumor, n = 217; benign tumor, n = 44; osteomyelitis of the maxilla or mandible, n = 41; cyst, n = 18; jaw deformity, n = 3; implant status, n = 2; and unidentified, n = 1. Paired sets of the initial and at least two follow-up CT examinations were included in analysis. All images were obtained between 2010 and 2019 at the Division of Oral and Maxillofacial Radiology, Kyushu Dental University Hospital, Japan. The mean (± standard deviation) follow-up period was 29 ± 18 months; range, 3–75 months. We judged that the patients’ informed consent was not required for this retrospective study. The study protocol was approved by the institutional review board (IRB) of Kyushu Dental University (No. 12-19).

CT was performed using an Activion 16 (Toshiba, Tokyo, Japan) scanner. Contiguous axial images of thickness 3 mm were obtained using standard algorithms and were viewed with soft-tissue windows.

Two experienced dental radiologists (K. Y. and M. O.) evaluated the images for the presence of tonsilloliths and each measured tonsillolith length, which was recorded as the mean value of the two measurements.

**Fig. 1:**
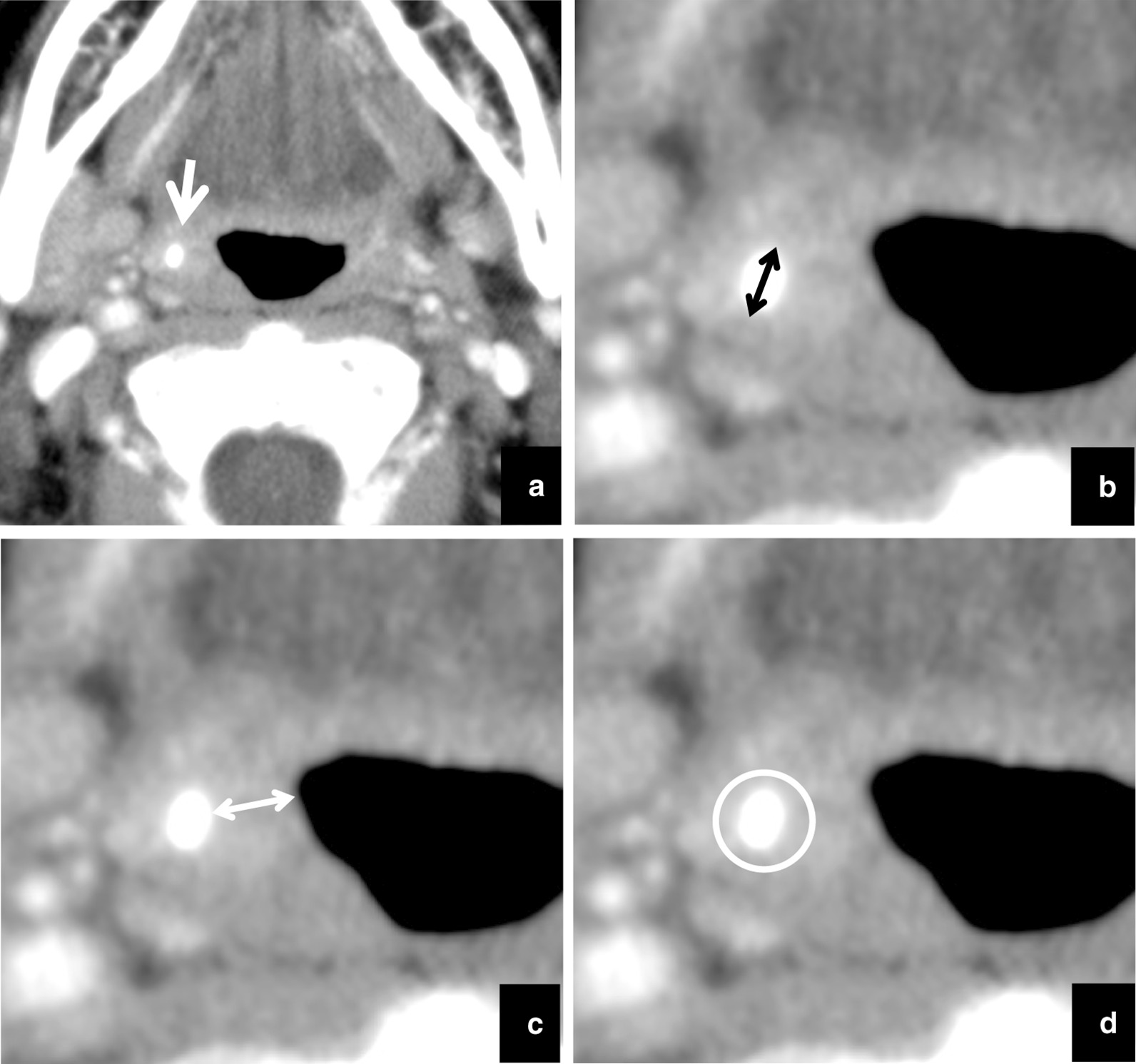
Methods for measurement of size, CT number, and depth of tonsilloliths on CT images. **a** The largest calculus (soft-tissue window setting, arrow) in the palatine tonsil was included in the analysis. **b** Size was defined as the long axis measurement (arrow) on soft-tissue window setting. **c** Position was defined as the shortest distance (arrow) from the surface of the respiratory tract on soft-tissue window setting. **d** Calcification level was defined as the maximum HU number on soft-tissue window setting, and the region of interest included the whole calculus (circle)

Tonsillolith pathology was diagnosed based on the system of Oda et al. [1]. Tonsilloliths were defined as structures of CT density > 100 Hounsfield units (HU). The following were recorded on the initial and follow-up scans: the number of masses or high-density structures within the palatine tonsil; in the case of multiple tonsilloliths, the size, position, and calcification level of the largest calculus were analyzed (Fig. 1a). Change in size was defined as difference in the long axis measurement between examinations on images with soft-tissue window setting (Fig. 1b). Change in position was defined as difference in depth with respect to the respiratory tract between examinations on images with soft-tissue window setting (Fig. 1c). Change in calcification level was defined as difference in maximum HU number between examinations on images with soft-tissue window setting (Fig. 1d). If a tonsillolith had disappeared on the follow-up CT examination, the last CT examination on which the tonsillolith was seen was used to assess change.

All statistical analyses were performed using IBM SPSS Statistics version 26 statistical software (SPSS, Chicago, Illinois, USA). Categorical variables were compared using the chi-square test. The relationships between categorical variables were assessed using Pearson’s correlation coefficient. Results were considered significant if *p* < 0.05.

## Results

### Initial examination

On the initial CT examination, at least one tonsillolith was detected in 134/326 patients (41.1%). In total, 290 tonsilloliths were detected in these 134 patients: 65 patients had one calculus, 32 had two, 17 had three, 6 had four, 9 had five, and 5 had six or more. The maximum number detected was 10.

### Follow-up examinations

The mean follow-up period was 20 ± 14 months; range, 3–71 months. The diagnoses followed up were malignant tumor, n = 217; benign tumor, n = 44; osteomyelitis of the maxilla or mandible, n = 41; cyst, n = 18; implant check, n = 2; jaw deformity, n = 2; facial fractures, n = 1; and unidentified, n = 1. There was no significant difference in the distribution of diseases that were followed up between patients with and without tonsilloliths (chi-square test, *p* = 0.357).

### Change in the number of tonsilloliths

Table 1 summarizes change in the number of tonsilloliths during the follow-up period. The number of tonsilloliths increased in 13/326 patients (4.0%) and decreased in 31/326 patients (9.5%) during the follow up period.

**Table 1:** Number of tonsilloliths and ratios of change in number during the follow-up period

Initial examination	Decrease		No change		Increase	
	n	Ratio (%)	n	Ratio (%)	n	Ratio (%)
0	–	–	187	97.5	5	2.5
1	15	23.8	49	74.6	1	1.6
2	5	15.2	25	75.8	2	9.1
3	4	26.7	12	73.3	1	0.0
4	2	33.3	3	50.0	1	16.7
5	4	57.1	5	42.9	0	0.0
6	1	50.0	0	0.0	1	50.0
7	0	0.0	1	50.0	1	50.0
10	0	0.0	0	0.0	1	100.0
Total	31	9.5	282	86.5	13	4.0

n = number of patients

### Change in tonsilloliths over time on CT

#### Size

**Fig. 2:**
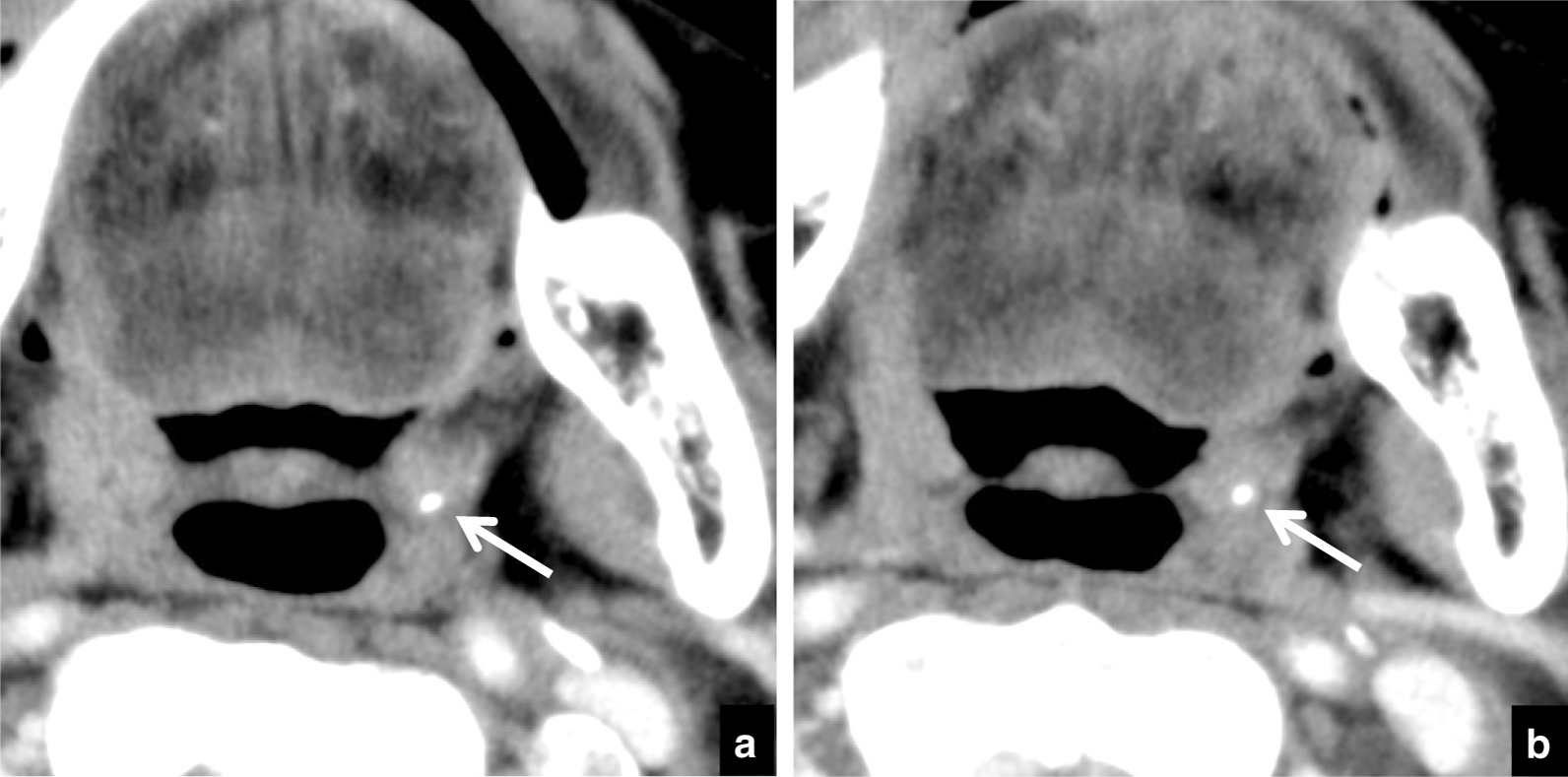
A 70-year-old subject with malignant tumor of the left mandibular gingiva in whom tonsillolith size showed no dynamic change on CT. **a** Axial CT image at the palatine tonsillar level obtained at the initial examination. A tonsillolith (arrow) is seen as a high-density dot. The long axis of the tonsillolith was 2.7 mm. **b** Follow-up CT image at the same level 14 months later. The long axis of the tonsillolith (arrow) was 2.7 mm, the same as at the initial examination

**Fig. 3:**
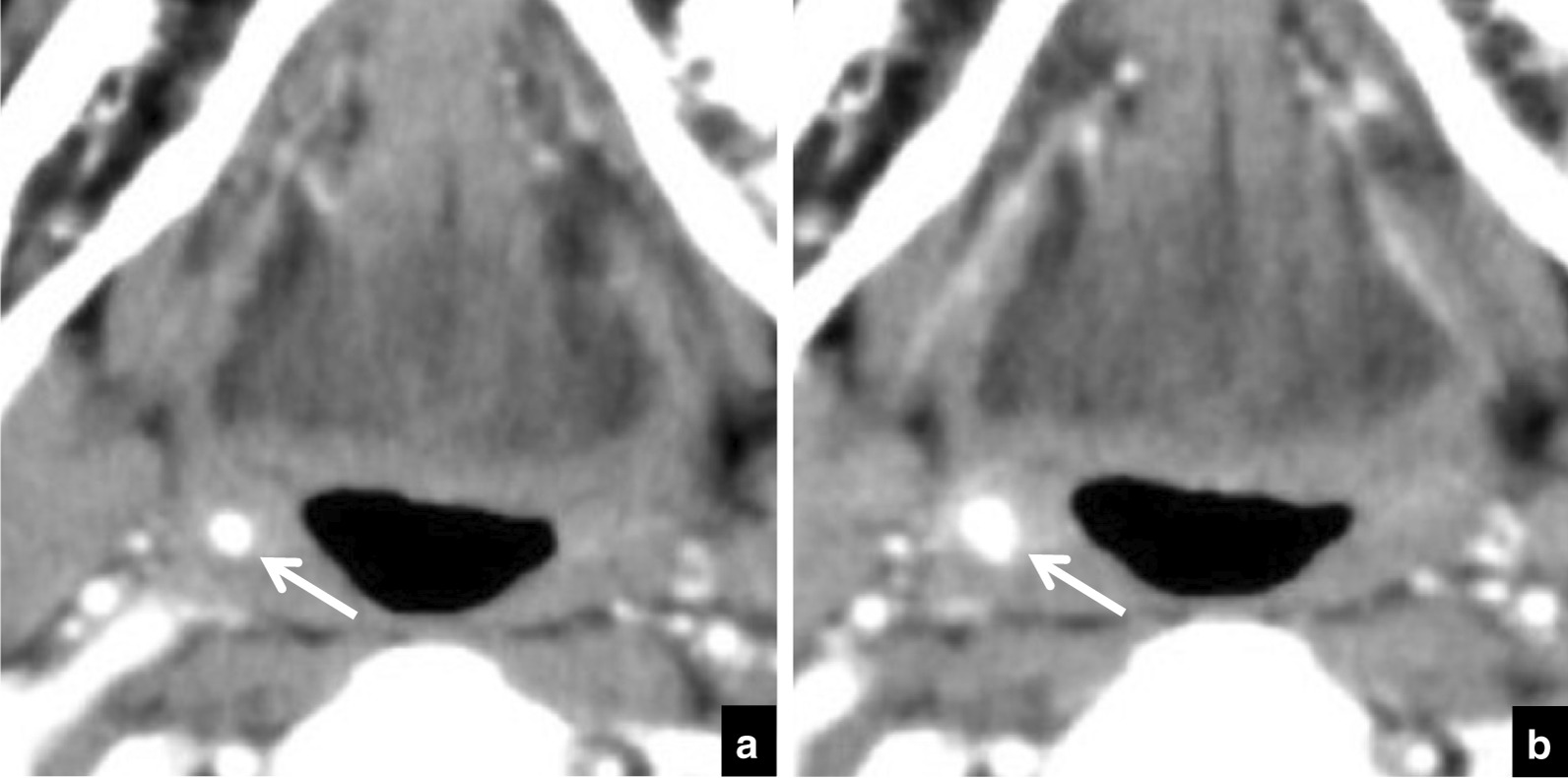
A 70-year-old subject with malignant tumor of the tongue in whom tonsillolith size increased on CT. **a** Axial CT images at the palatine tonsillar level at the initial examination. The arrow indicates a tonsillolith. The long axis of the tonsillolith was 1.4 mm. **b** Follow-up CT image at the same level 42 months later. The long axis of the tonsillolith (arrow) has increased to 2.4 mm

**Fig. 4:**
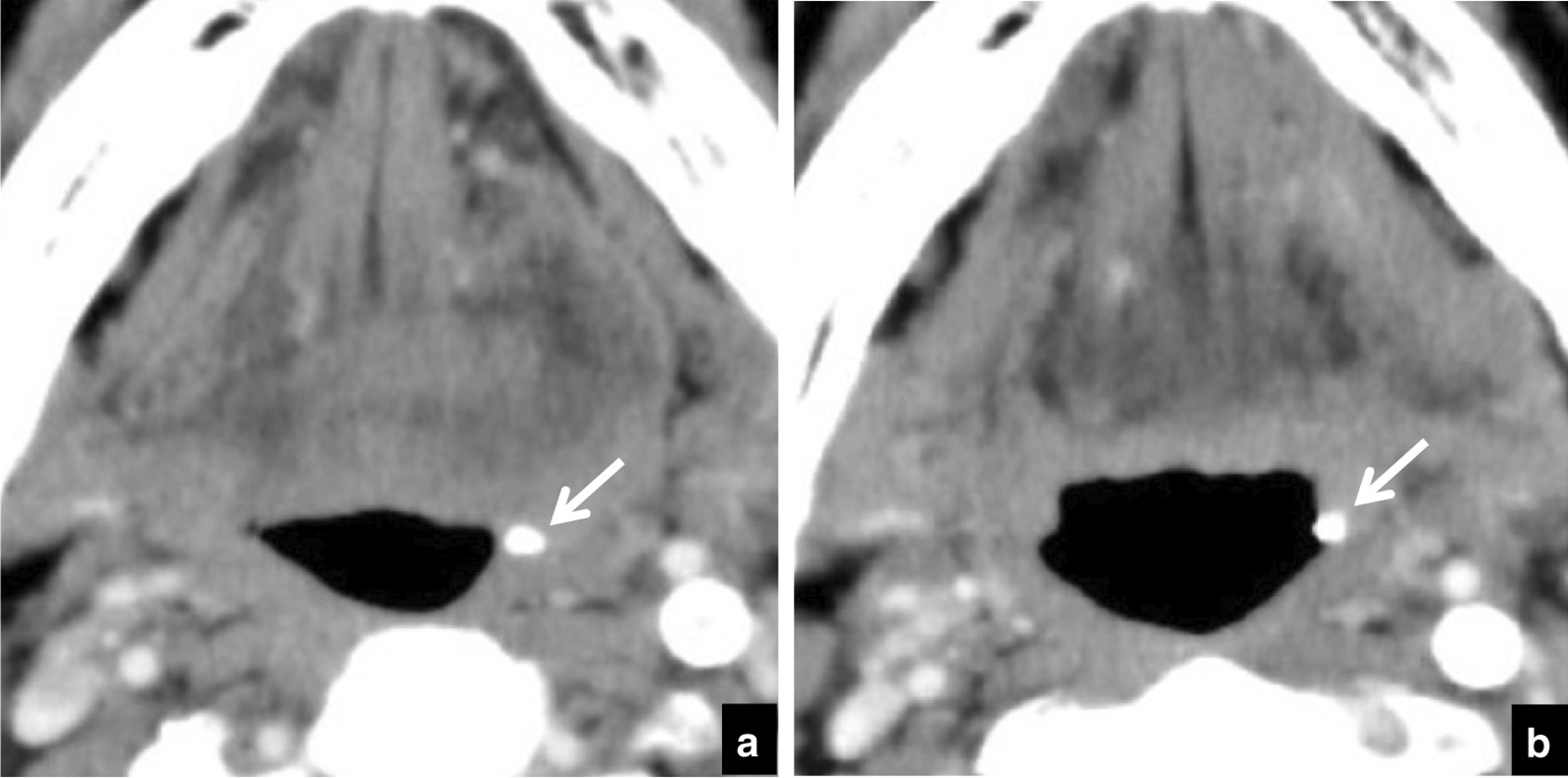
A 60-year-old subject with malignant tumor of the tongue in whom tonsillolith size decreased on CT. **a** Axial CT images at the palatine tonsillar level at the initial examination. The arrow indicates a tonsillolith. The long axis of the tonsillolith is 5.2 mm. **b** Follow-up CT image at the same level 18 months later. The long axis of the tonsillolith (arrow) has decreased to 4.3 mm

Table 2 lists the distributions of tonsillolith sizes and the ratios of change in size. In 99/134 patients with tonsilloliths, there was no change in size during the follow-up period (Fig. 2). In 35/134 patients, tonsillolith size changed during the follow-up period: 34/35 tonsilloliths increased in size (Fig. 3) and 1/35 decreased in size (Fig. 4). The mean change in size was 0.25 ± 0.51 mm; range, − 0.84 to 2.81 mm. The mean rate of accretion in the 134 tonsilloliths was 0.15 ± 0.36 mm/year; range, − 1.22 to 1.96 mm/year. In the 34 tonsilloliths that increased in size, mean change was 1.01 ± 0.45 mm; range, 0.54–2.81 mm; and mean accretion rate was 0.61 ± 0.41 mm/year; range, 0.15–1.96 mm/year. The one tonsillolith that grew smaller decreased by − 1.22 mm/year (− 0.84 mm/251 days).

**Table 2:** Distribution of tonsillolith size and ratios of change in size during the follow-up period

Size on initial CT (mm)	Increase		No change		Decrease		Total number
	N	Ratio (%)	n	Ratio (%)	n	Ratio (%)	
< 1.0	2	66.7	1	33.3	0	0.0	3
1.0–1.5	8	29.6	19	70.4	0	0.0	27
1.5–2.0	12	25.0	36	75.0	0	0.0	48
2.0–2.5	6	35.3	11	64.7	0	0.0	17
2.5–3.0	2	14.3	12	85.7	0	0.0	14
3.0–3.5	4	36.4	7	63.6	0	0.0	11
3.5–4.0	0	0.0	8	100.0	0	0.0	8
4.0–4.5	0	0.0	1	100.0	0	0.0	1
4.5–5.0	0	0.0	4	100.0	0	0.0	4
5.0–5.5	0	0.0	0	0.0	1	100.0	1
Total	34	25.4	99	73.9	1	0.7	134

n = number of patients

#### Position

**Fig. 5:**
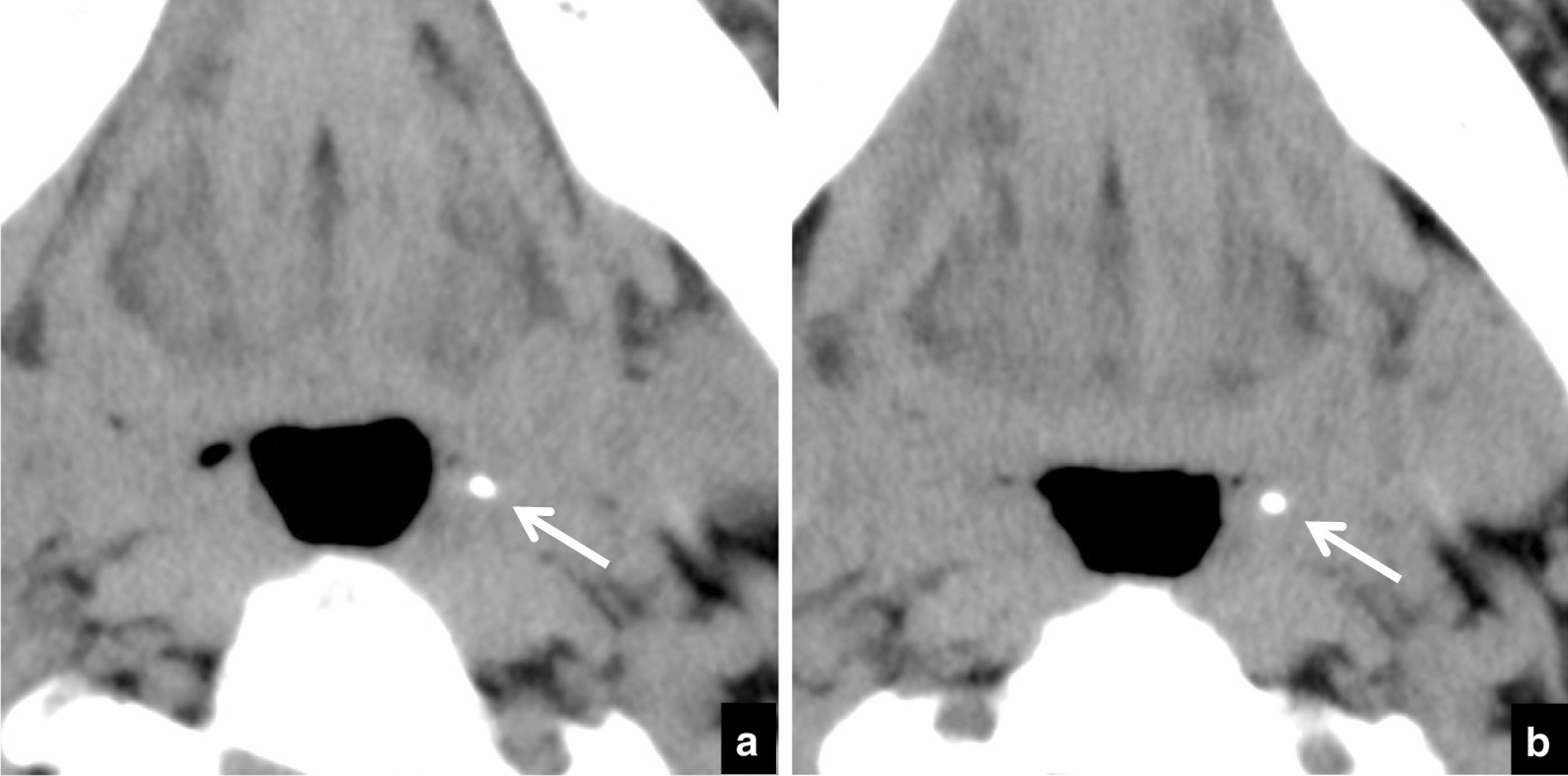
A 70-year-old subject with malignant tumor of the tongue in whom tonsillolith size showed no dynamic movement on CT. **a** Axial CT images at the palatine tonsillar level at the initial examination. The depth of the tonsillolith (arrow) is 5.0 mm. **b** Follow-up CT image at the same level 7 months later. The depth of the tonsillolith (arrow) is 5.0 mm, the same as at the initial examination

**Fig. 6:**
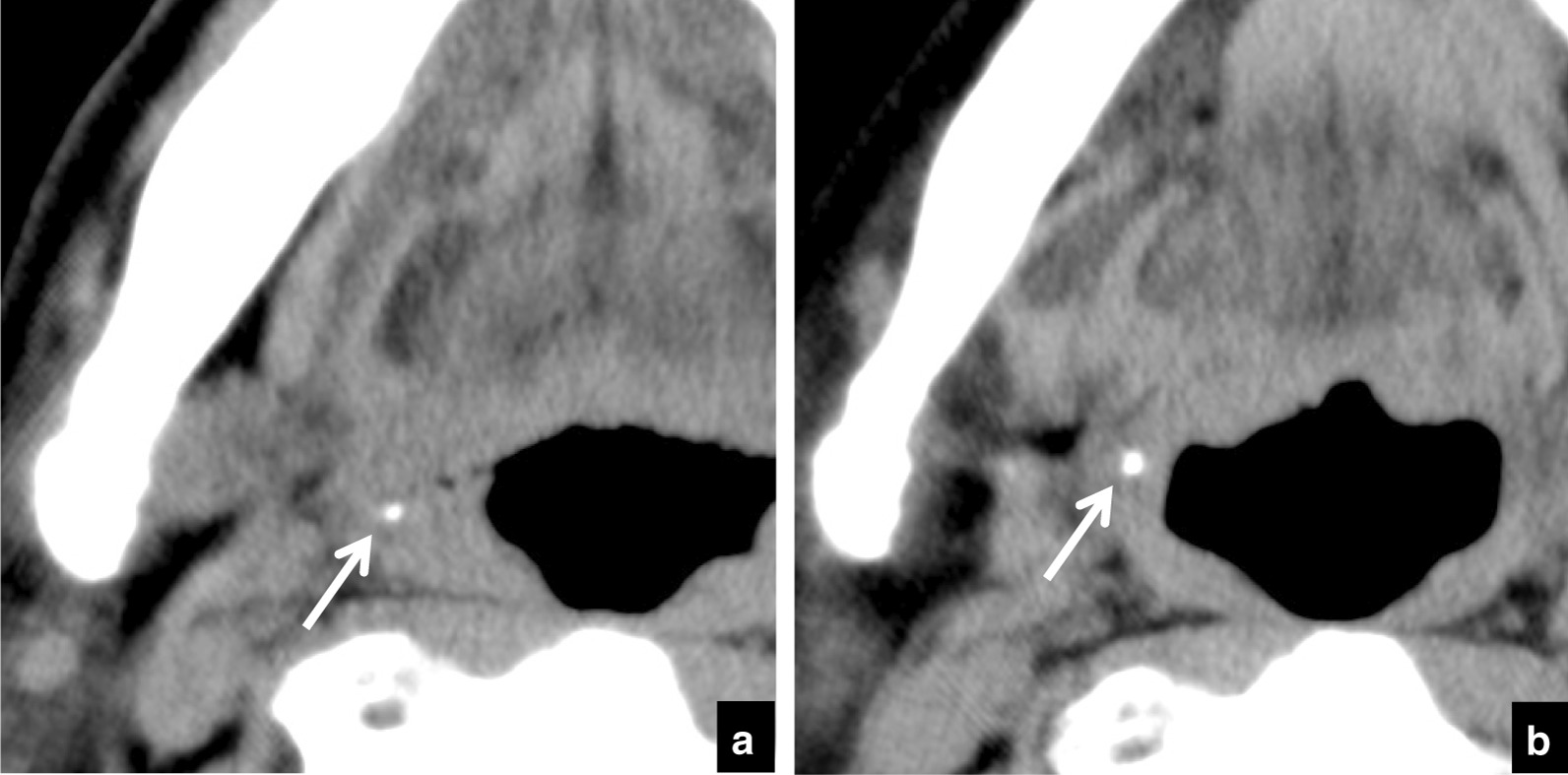
A 70-year-old subject with osteomyelitis of right mandibular gingiva in whom dynamic movement of the tonsillolith was seen toward the internal side on CT. **a** Axial CT image at the palatine tonsillar level at the initial examination. The depth of the tonsillolith (arrow) is 6.1 mm. **b** Follow-up CT image at the same level 48 months later. The depth of the tonsillolith (arrow) is now 3.3 mm, following movement in the medial direction (toward the respiratory tract)

**Fig. 7:**
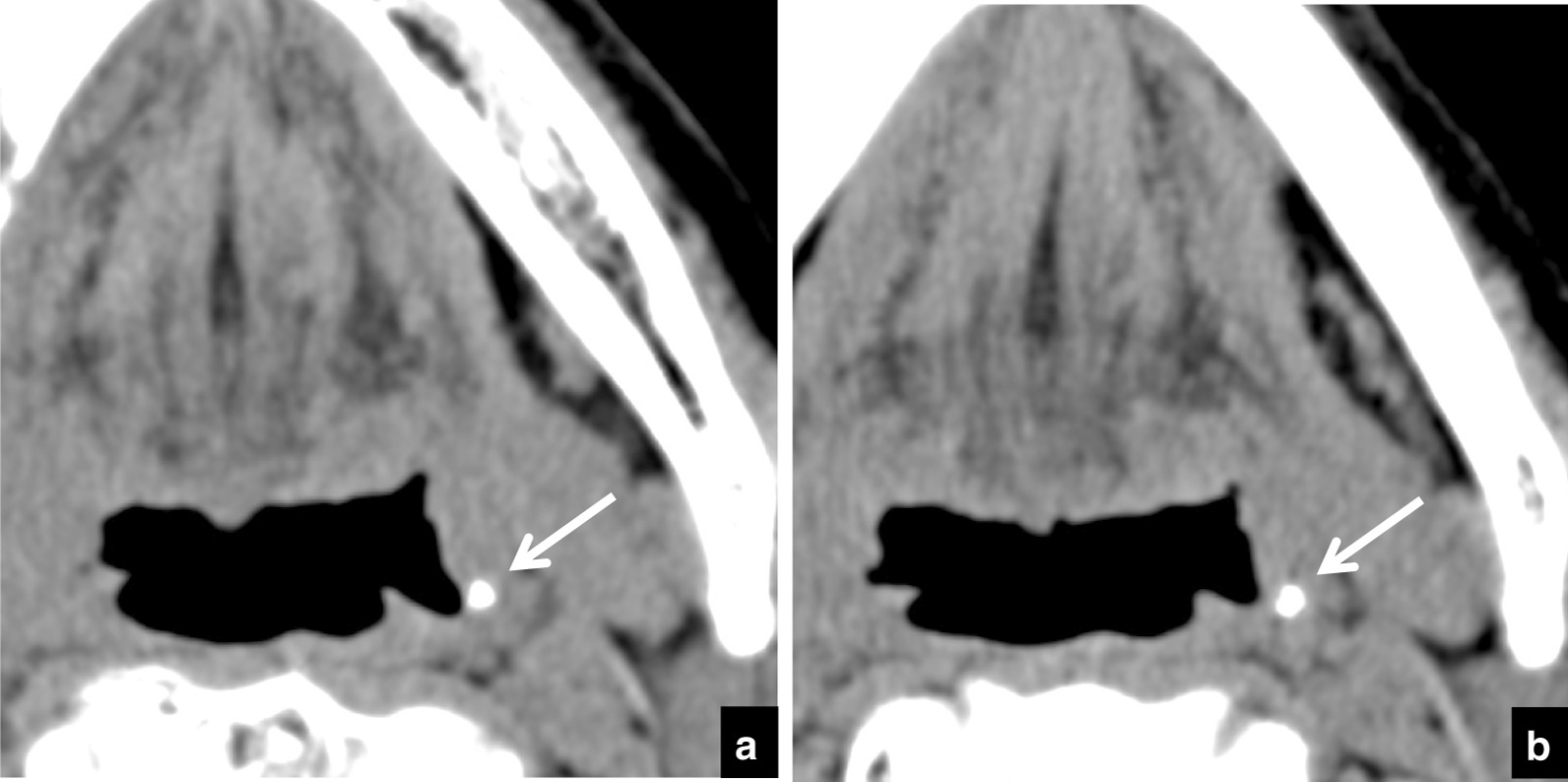
An 80-year-old subject with malignant tumor of the right mandible in whom dynamic movement of the tonsillolith was seen toward the external side on CT. **a** Axial CT image at the palatine tonsillar level at the initial examination. The depth of the tonsillolith (arrow) is 0.7 mm. **b** Follow up CT image at the same level 6 months later. The depth of the tonsillolith (arrow) is now 1.8 mm, following movement in the lateral direction (away from the respiratory tract)

Table 3 lists the distributions of depths and the ratios of change in depth. During the follow-up period a change in position was seen in 50 tonsilloliths and there was no change in 84 (Fig. 5). Most movement (in 46/50) was in the medial direction (toward the respiratory tract) (Fig. 6). Movement was in the lateral direction (away from the respiratory tract) in 4 patients (Fig. 7). In the 134 patients with tonsilloliths, mean change in position was − 0.51 ± 1.04; range, from − 4.92 to 1.49 mm; and the mean rate of movement was − 0.42 ± 1.23; range, from − 7.17 to 3.97 mm/year. In the 46 tonsilloliths that moved medially, the mean distance was − 1.58 ± 1.14 mm; range, from − 4.92 to − 0.52 mm; and the mean rate of movement was − 1.38 ± 1.59 mm/year; range, from − 7.17 to − 0.14 mm/year. In the 4 tonsilloliths that moved laterally, mean distance was 1.03 ± 0.35 mm; range, 0.70–1.49 mm; and the mean rate of movement was 1.82 ± − 1.73 mm/year; range, 0.40–3.97 mm/year.

**Table 3:** Distribution of tonsillolith depth and ratios of change in depth during the follow-up period

Depth on initial CT (mm)	Medial movement		No change		Lateral movement		Total
	n	Ratio (%)	n	Ratio (%)	n	Ratio (%)	
0	0	0.0	29	100.0	0	0.0	29
0–1.0	0	0.0	2	66.7	1	33.3	3
1.0–2.0	5	17.2	24	82.8	0	0.0	29
2.0–3.0	5	35.7	9	64.3	0	0.0	14
3.0–4.0	6	60.0	4	40.0	0	0.0	10
4.0–5.0	6	54.5	4	36.4	1	9.1	11
5.0–6.0	5	55.6	4	44.4	0	0.0	9
6.0–7.0	4	80.0	1	20.0	0	0.0	5
7.0–8.0	4	66.7	1	16.7	1	16.7	6
8.0–9.0	1	50.0	1	50.0	0	0.0	2
9.0–10.0	0	0.0	1	100.0	0	0.0	1
10.0–11.0	5	83.3	1	16.7	0	0.0	6
11.0–12.0	2	50.0	1	25.0	1	25.0	4
12.0 <	3	60.0	2	40.0	0	0.0	5
Total	46	34.3	84	62.7	4	3.0	134

n = number of patients

#### Calcification level

Table 4 lists the distributions of HU numbers and the ratios of change in HU number. HU number increased in 113/134 tonsilloliths, was unchanged in 4/134, and decreased in 17/134. Mean change in HU number in the 134 patients with tonsilloliths was 58.3 ± 87.2 HU; range, from − 109 to 490 HU. The mean rate of change in HU number in the 134 patients with tonsilloliths was 51.6 ± 102.1 HU/year; range, from − 267.0 to 612.4 HU/year. In the 113 tonsilloliths that increased in HU number, mean degree of increase in HU number was 73.8 ± 85.8 HU; range, 1–490 HU. In the 17 tonsilloliths that decreased in size, mean HU number was − 30.7 ± 28.0 HU; range, from − 109 to − 1 HU. The mean rate of HU number increase in the 113 tonsilloliths was 63.8 ± 96.3 HU/year; range, 0.45–612.4 HU/year; and the mean rate of HU number decrease in the 17 tonsilloliths was − 38.4 ± 66.8 HU/year; range, from − 267 to − 0.4 HU/year.

**Table 4:** Distributions of CT numbers in tonsilloliths and ratios of change in CT number during the follow-up period

CT number on initial CT (HU)	Increase in CT number		No change		Decrease in CT number		Total number
	n	Ratio (%)	n	Ratio (%)	n	Ratio (%)	
< 200	52	89.7	2	3.4	4	6.9	58
200–300	23	85.2	0	0.0	4	14.8	27
300–400	15	75.0	0	0.0	5	25.0	20
400–500	7	77.8	2	22.2	0	0.0	9
500–600	6	85.7	0	0.0	1	14.3	7
600–700	4	100.0	0	0.0	0	0.0	4
700–800	1	100.0	0	0.0	0	0.0	1
800–900	2	66.7	0	0.0	1	33.3	3
900–1000	1	33.3	0	0.0	2	66.7	3
1000 <	2	100.0	0	0.0	0	0.0	2
Total	113	84.3	4	3.0	17	12.7	134

n = number of patients, HU = Hounsfield units

### Relationship between CT findings and change in characteristics

A significant correlation was found between initial size and the annual rate of change in size (Pearson’s correlation coefficient: r = − 0.174, *p* = 0.045). No significant correlations were found between initial size and change in depth (Pearson’s correlation coefficient: r = 0.090, *p* = 0.300) or HU number (Pearson’s correlation coefficient: r = − 0.029, *p* = 0.739). A significant correlation was found between initial depth and annual rate of change in depth (Pearson’s correlation coefficient: r = − 0.279, *p* = 0.001). No significant correlations were found between initial depth and change in size (Pearson’s correlation coefficient: r = 0.146, *p* = 0.093) or HU number (Pearson’s correlation coefficient: annual change in position r = − 0.001, *p* = 0.992). Regarding HU number, no significant correlation was found against annual change in size (Pearson’s correlation coefficient: r = − 0.127, *p* = 0.143), depth (Pearson’s correlation coefficient: r = 0.105, *p* = 0.225), or HU number (Pearson’s correlation coefficient: r = − 0.020, *p* = 0.822).

## Discussion

We hypothesized that there would be dynamic change in the size, position, and calcification level of tonsilloliths and a greater tonsillolith detection rate as time proceeds. The results revealed dynamic fluctuation of the calcification levels of all tonsilloliths, as well as a tendency for the tonsilloliths to be excreted from the body with increasing size of the tonsillolith. The active dynamics of tonsilloliths function to remove foreign matter from the body.

The detection rates of dynamic change in terms of size and position were only 26.1% (35/134 patients) and 37.3% (50/134 patients), respectively. Regarding tonsillolith size, mean change was 0.15 ± 0.36 mm/year; range, from − 1.22 to 1.96 mm/year. To the best of our knowledge, there is no previous report of the rate of increase of size of tonsilloliths; therefore, our data contribute to knowledge in this field. Of note in the present study, large tonsilloliths detected in the oropharyngeal area in asymptomatic subjects showed no change in size over the course of one year. However, a significant correlation was found between initial size and the annual rate of change in size; the smaller the tonsillolith detected on CT, the larger the annual rate of change in size. It is important to pay attention to small tonsilloliths because they have potential to increase in size as an active focus. It is likely that larger tonsilloliths did not change in position because of their size. In contrast, a significant correlation was found between depth and the annual rate of change of depth. The deeper the tonsillolith on the CT image, the greater was the annual rate of negative change in depth (movement toward the respiratory tract). This finding indicates that tonsilloliths in a more lateral position (away from the respiratory tract) can potentially move a greater distance toward the respiratory tract. In most tonsilloliths, the direction of movement was mainly medial (toward the respiratory tract). Tonsilloliths were spontaneously excreted from the body as they grew in size, as a system for removal of foreign matter. Of note, dynamic change in size and position may damage the surrounding tissue and circulation. We hypothesize that patients with tonsilloliths that undergo dynamic change may have increased risk of tonsillar abscess and halitosis for this reason. Further study is necessary to clarify this hypothesis.

An unexpected finding was the dynamic change in calcification level of almost all tonsilloliths (97%) during the follow-up period. HU number increased in 84.3% of these tonsilloliths, but decreased in 12.7%. The present results suggest that the calcification level of tonsilloliths might change constantly, for the possible reason that tonsilloliths are continuously exposed to high levels of calcium ions in the saliva [14]. Accordingly, calcium may be deposited as dystrophic calcifications, thus increasing the HU number of large tonsilloliths. The mean rate of calcification increase was 63.8 ± 96.3 HU/year; range, 0.45–612.4 HU/year. Unexpectedly, there was a low rate of decalcification in tonsilloliths. The mean rate of HU decrease in 17 tonsilloliths was − 38.4 ± 66.8 HU/year; range, from − 267 to − 0.35 HU/year. It is unclear why so few tonsilloliths decalcified. The dynamic fluctuation of the calcification levels of all tonsilloliths indicate that tonsillolith calcification may always be in a very active phase. An unknown mechanism might affect dynamic change in tonsilloliths. Further studies are required to clarify the reason for the low rate of decalcification.

Limitations of the present study are the small sample size, and that the data were obtained from subjects with diseases mainly involving oropharyngeal area, rather than the oral and maxillofacial region. Thus, the present results should be interpreted as being relevant to relatively healthy, active populations. A further limitation is that only Japanese subjects were examined. We did not assess the presence and characteristics of tonsilloliths on digital panoramic radiographs because tonsilloliths can be visualized only on 5.7–7.3% of these images [1, 9].

## Conclusions

The present evaluation of tonsillolith dynamics found that the number of tonsilloliths increased and decreased, and the size and position of tonsilloliths changed during the follow-up period. These processes were very active in all tonsilloliths. The tendency for tonsilloliths to be excreted from the body as their size increased indicates a gradual process of removal of foreign matter from the body.

## Data Availability

All the datasets used and analyzed during the current study are available from the corresponding author on reasonable request.

## Abbreviations

CT: Computed tomography
HU: Hounsfield units

## References

[1] Oda M, Kito S, Tanaka T, Nishida I, Awano S, Fujita Y, Saeki K, Matsumoto-Takeda S, Wakasugi-Sato N, Habu M, Kokuryo S, Kodama M, Kaneuji T, Yoshiga D, Miyamoto I, Nishimura S, Yamashita Y, Maki K, Tominaga K, Yoshioka I, Ansai T, Morimoto Y (2013). Prevalence and imaging characteristics of detectable tonsilloliths on 482 pairs of consecutive CT and panoramic radiographs. BMC Oral Health.

[2] Takahashi A, Sugawara C, Kudoh T, Ohe G, Takamaru N, Tamatani T, Nagai H, Miyamoto Y (2017). Prevalence and imaging characteristics of palatine tonsilloliths evaluated on 2244 pairs of panoramic radiographs and CT images. Clin Oral Investig.

[3] Kim MJ, Kim JE, Huh KH, Yi WJ, Heo MS, Lee SS, Choi S (2018). Multidetector computed tomography imaging characteristics of asymptomatic palatine tonsilloliths: a retrospective study on 3886 examinations. Oral Surg Oral Med Oral Pathol Oral Radiol.

[4] Missias EM, Nascimento E, Pontual M, Pontual AA, Freitas DQ, Perez D, Ramos-Perez F (2018). Prevalence of soft tissue calcifications in the maxillofacial region detected by cone beam CT. Oral Dis.

[5] Takahashi A, Sugawara C, Kudoh K, Yamamura Y, Ohe G, Tamatani T, Miyamoto Y (2018). Lingual tonsillolith: prevalence and imaging characteristics evaluated on 2244 pairs of panoramic radiographs and CT images. Dentomaxillofac Radiol.

[6] Rio AC, Franchi-Teixeira AR, Nicola EM (2008). Relationship between the presence of tonsilloliths and halitosis in patients with chronic caseous tonsillitis. Br Dent J.

[7] Caldas MP, Neves EG, Manzi FR, de Almeida SM, Bóscolo FN, Haiter-Neto F (2007). Tonsillolith—report of an unusual case. Br Dent J.

[8] Ansai T, Takehara T (2005). Tonsillolith as a halitosis-inducing factor. Br Dent J.

[9] Sutter W, Berger S, Meier M, Kropp A, Kielbassa AM, Turhani D (2018). Cross-sectional study on the prevalence of carotid artery calcifications, tonsilloliths, calcified submandibular lymph nodes, sialoliths of submandibular gland, and idiopathic osteosclerosis using digital panoramic radiography in a Lower Austrian subpopuation. Quintessence Int.

[10] Mesolella M, Cimmino M, Di Martino M, Criscuoli G, Albanese L, Galli V (2004). Tonsillolith. Case report and review of the literature. Acta Otorhinolaryngol Ital.

[11] Weller CV (1924). The incidence and pathogenesis of tonsillar concretions. Ann Otol Rhinol Laryngol.

[12] Aspestrand F, Kolbenstvedt A (1987). Calcifications of the palatine tonsillary region: CT demonstration. Radiology.

[13] Fauroux MA, Mas C, Tramini P, Torres JH (2013). Prevalence of palatine tonsilloliths: a retrospective study on 150 consecutive CT examinations. Dentomaxillofac Radiol.

[14] de Almeida PV, Grégio AM, Machado MA, de Lima AA, Azevedo LR (2008). Saliva composition and functions: a comprehensive review. J Contemp Dent Pract.

